# Impact of Hypothermic Oxygenated Machine Perfusion on Hepatocellular Carcinoma Recurrence after Liver Transplantation

**DOI:** 10.3390/jpm13050703

**Published:** 2023-04-22

**Authors:** Federica Rigo, Nicola De Stefano, Damiano Patrono, Victor De Donato, Ludovico Campi, Diana Turturica, Teresa Doria, Veronica Sciannameo, Paola Berchialla, Francesco Tandoi, Renato Romagnoli

**Affiliations:** 1 General Surgery 2U—Liver Transplant Unit, Azienda Ospedaliero Universitaria Città della Salute e della Scienza di Torino, 10126 Turin, Italy; 2 Centre for Biostatistics, Epidemiology and Public Health (C-BEPH), Department of Clinical and Biological Sciences, University of Torino, 10126 Turin, Italy; 3 HPB and Liver Transplant Unit, Azienda Ospedaliero Universitaria Consorziale Policlinico, 70124 Bari, Italy

**Keywords:** hypothermic oxygenated machine perfusion, hepatocellular carcinoma, tumor recurrence, ischemia/reperfusion injury

## Abstract

Background: Machine perfusion may be able to mitigate ischemia-reperfusion injury (IRI), which increases hepatocellular carcinoma (HCC) recurrence after liver transplantation (LT). This study aimed to investigate the impact of dual-hypothermic oxygenated machine perfusion (D-HOPE) on HCC recurrence in LT. Methods: A single-center retrospective study was conducted from 2016 to 2020. Pre- and postoperative data of HCC patients undergoing LT were analyzed. Recipients of a D-HOPE-treated graft were compared to those of livers preserved using static cold storage (SCS). The primary endpoint was recurrence-free survival (RFS). Results: Of 326 patients, 246 received an SCS-preserved liver and 80 received a D-HOPE-treated graft (donation after brain death (DBD), n = 66; donation after circulatory death (DCD), n = 14). Donors of D-HOPE-treated grafts were older and had higher BMI. All DCD donors were treated by normothermic regional perfusion and D-HOPE. The groups were comparable in terms of HCC features and estimated 5-year RFS according to the Metroticket 2.0 model. D-HOPE did not reduce HCC recurrence (D-HOPE 10%; SCS 8.9%; *p* = 0.95), which was confirmed using Bayesian model averaging and inverse probability of treatment weighting-adjusted RFS analysis. Postoperative outcomes were comparable between groups, except for lower AST and ALT peak in the D-HOPE group. Conclusions: In this single-center study, D-HOPE did not reduce HCC recurrence but allowed utilizing livers from extended criteria donors with comparable outcomes, improving access to LT for patients suffering from HCC.

## 1. Introduction

Hepatocellular carcinoma (HCC) is the most common primary liver malignancy, accounting for approximately 80–90% of all liver cancers [1]. Despite advances in liver resection surgery, liver transplantation (LT), chemotherapy, and locoregional therapies, the overall prognosis of HCC remains poor, with overall recurrence rates ranging from 50% to 70% within 5 years after initial treatment [1]. LT remains the best curative option for patients affected by HCC, addressing both the tumor and the underlying chronic liver disease. The use of the Milan criteria (MC) to determine eligibility for LT in HCC patients first led to a significant improvement in survival rates of LT for HCC [2]. More recently, scores incorporating alpha fetoprotein (AFP) levels as a surrogate of tumor biology have been proposed to better assess patients eligibility for LT [3,4,5,6,7,8].

Due to donor shortage, organs from extended criteria donors (ECD) are being increasingly utilized and are most frequently allocated to patients with HCC with normal hepatic function. ECD definition is broad [9] but mostly includes donation after circulatory death (DCD), elderly donors, or liver grafts with significant macrovesicular steatosis. Although the use of ECD allows expanding donor pool, these organs have inferior tolerance to ischemia/reperfusion injury (IRI) [10]. In animal experiments, IRI has been strongly associated with tumor recurrence, as it establishes a local microenvironment that supports tumor cell invasion, migration, and growth [11,12,13,14,15,16,17,18,19,20,21,22,23,24,25,26,27,28,29,30].

Machine perfusion techniques have been reintroduced in clinical practice to improve graft preservation, extend preservation time, and allow viability assessment [31,32,33,34,35,36,37,38,39,40,41,42]. Among machine perfusion modalities, hypothermic oxygenated perfusion (HOPE) has been shown to mitigate IRI in ECD grafts and to improve postoperative outcomes [40,41,43,44,45,46,47,48,49,50,51,52,53].

By mitigating IRI, HOPE could potentially reduce the risk of HCC recurrence linked to ECD utilization [54]. However, the clinical evidence supporting this hypothesis is limited to one retrospective study [55], stressing the need for additional research. Thus, the aim of this single-center study was to investigate the impact of dual-HOPE (D-HOPE) on HCC recurrence after LT.

## 2. Materials and Methods

### 2.1. Study Popolation and Design

We conducted a single-center retrospective cohort study including adult (age ≥ 18) patients with HCC who underwent deceased donor LT at our center between January 2016 and December 2020, to compare HCC recurrence according to the preservation method (SCS versus D-HOPE). The study was conducted according to the principles of the Helsinki and Istanbul declarations and was approved by the ethics committee of our institution. The study period was decided to allow for a minimum follow-up of 2 years after LT. Patients who died intraoperatively, with other tumor types at explant pathology (e.g., incidental cholangiocarcinoma or hepatocholangiocarcinoma) or no residual HCC in the absence of pre-LT downstaging were excluded. Patients receiving a liver treated with perfusion techniques other than D-HOPE were also excluded. The primary endpoint was recurrence-free survival. Secondary endpoints were measures of postoperative outcomes, surgical complications, and incidence of biopsy-proven acute cellular rejection. Outcome measures included peak transaminase levels, early allograft dysfunction (EAD) rate, acute kidney injury (AKI) rate and severity, hospital and ICU stay, and postoperative complications including biliary and anastomotic complications. EAD and AKI were defined according to Olthoff et al. [56] and KDIGO guidelines [57], respectively. Liver graft assessment following transplantation score (L-GrAFT) was used as a measure of post-LT graft function [58]. Postoperative complications were graded using the Clavien–Dindo classification [59], which was also used to calculate the comprehensive complication index (CCI) [60]. Histological preservation injury was assessed on time-0 biopsies, which were systematically obtained at the end of LT operation [61]. Biliary complications [62] were diagnosed based on the 3-month cholangiogram obtained before T-tube removal or by using magnetic resonance cholangiopancreatography if clinically indicated.

Data on tumor burden (number of nodes, maximum and total node diameter, AFP values) at diagnosis, listing, and transplantation; downstaging procedures (type and number); explant pathology report (number of nodes, grading, micro/macrovascular invasion, and degree of necrosis); and follow-up data (including immunosuppressive regimens) were prospectively collected and retrospectively analyzed. Baseline recipient (biometric characteristics, comorbidities, MELD score) and donor (donor type, D-MELD, donor risk index—DRI) [63] features, as well as procedural variables, were also recorded.

### 2.2. Patient Management

The D-HOPE protocol was applied as described elsewhere [49,50]. Briefly, livers in both groups underwent an initial period of SCS using Celsior solution (IGL, Lissieux, France). Grafts from DCD donors were procured after a period of normothermic regional perfusion, as previously reported [48,64]. The use of D-HOPE was systematic in grafts from DCD donors and was considered on a case-by-case basis in grafts from extended-criteria DBD donors, considering donor and recipient characteristics, expected preservation time, and donor-recipient matching [49]. Livers in the end-ischemic D-HOPE group were prepared on the backtable upon arrival at our transplant center and underwent a minimum of 90 min D-HOPE during recipient hepatectomy. D-HOPE was performed using the LiverAssist device (XVIVO, Goteborg, Sweden) primed with 3 L of Belzer MP solution (BridgeToLife, Northbrook, IL, USA) setting the pressure at 3–5 mmHg in the portal vein and at 25 mmHg in the hepatic artery. D-HOPE was not used for evaluation purposes, and all accepted grafts were eventually transplanted. Livers in both groups were flushed with chilled 5% albumin before implantation into the recipient.

Post-LT management was similar in both groups. Initial immunosuppression schedule included basiliximab as an induction, tacrolimus, steroids, and mycophenolate mofetil. Steroids were tapered and discontinued 3 months after LT. The introduction of everolimus was considered at 1-month follow-up in all patients without significant proteinuria or dyslipidemia. In the absence of worrisome symptoms, the surveillance of HCC recurrence included AFP levels every 3 months and a thoracoabdominal CT scan every 6 months for the first 2 years after LT. Further imaging exams were obtained if clinically indicated. The same surveillance protocol was applied to all patients regardless of HCC stage or preservation method.

### 2.3. Statistical Analysis

Categorical variables were expressed as counts and percentage, whereas continuous variables were expressed as median with interquartile range (IQR). Non-parametric Mann–Whitney test was used to compare continuous variables, whereas Chi-square or Fisher’s exact test were used for categorical variables, as appropriate. The impact of machine perfusion (MP) on HCC recurrence was evaluated both in terms of the recurrence rate and as a time-dependent variable using the proportional hazards Cox method. RFS according to the preservation method was analyzed using Kaplan–Meier curves and compared with the log-rank test. To account for potential confounding factors, we used inverse probability of treatment weighting (IPTW), which weights the data to balance baseline characteristics between the study groups, to adjust survival analysis [65]. Specifically, we estimated the propensity score for receiving D-HOPE versus SCS using a logistic regression model that included the following recipient and donor characteristics as covariates: donor age, donor BMI, macrovesicular steatosis, cold ischemia time, recipient age, HCC grading, and microvascular invasion. We then used the inverse of the propensity score as the weight for each patient in the analysis. The IPTW-adjusted Cox proportional hazards model was used to estimate the hazard ratio for HCC recurrence between the D-HOPE and SCS groups, with a robust variance estimator to account for the weighted data. A further analysis using Bayesian model averaging (BMA) was conducted on the entire cohort [66]. BMA is an approach that allows for the averaging of all possible statistical models supported by the data to obtain an unbiased estimate of the effect size of the different variables on the analyzed endpoint. The percentage of inclusion (PI) indicates the percentage of all possible models in which the variable has been included. The number of possible models is determined by all possible combinations of baseline covariates that have a posterior model probability of at least 1/20. PI can be directly interpreted as the probability of a variable being included in the model and indicates which variables have a confounding effect on the outcome and should be used for adjustment. The effect on the outcome is expressed as a hazard ratio (HR), while the probability of direction, defined as the probability that the HR is more than 1, is provided as a measure of the strength of the association.

Statistical significance (p) was set at a value of 0.05. Analyses were performed using R version 4.2.3 (R Foundation for Statistical Computing, Vienna, Austria). Bayesian model averaging and adjusted RFS analysis were performed using the packages “BMA: Bayesian Model Averaging” and “adjustedCurves: A comparison of different methods to adjust survival curves for confounders”.

## 3. Results

Some 398 patients underwent LT for HCC during the study period. Of these, 36 patients were excluded due to intraoperative death (n = 2, 0.6%), unconfirmed HCC at explant pathology (n = 20, 5.5%), incidental cholangiocarcinoma (n = 11, 3.0%), or the use of perfusion techniques other than D-HOPE (n = 3, 0.8%). Thus, 362 patients undergoing LT for HCC with a minimum follow-up of two years were included in the study cohort. Among them, 246 received grafts preserved using SCS, whereas the remaining 80 patients underwent transplantation with grafts treated with end-ischemic D-HOPE (DBD, n = 66; DCD, n = 14).

The HCC recurrence rate in the whole cohort was 9.2% (30 out of 326 patients), with a median time to recurrence from LT of 14 months (IQR: 11.2–23). Among those who experienced recurrence, 9 patients (30%) had intrahepatic recurrence, while 21 patients (70%) had extrahepatic recurrence. The pattern of HCC recurrence was comparable between study groups. In the D-HOPE group, 3 (37.5%) and 5 (62.5%) patients developed liver-only and systemic recurrence, whereas in the SCS group the proportions were 6 (27.3%) and 16 (72.7%), respectively (Fisher exact test, *p* = 0.92).

Baseline patient and donor characteristics, operational details, HCC features, and immunosuppression regimen data are summarized in Table 1.

Preferential use of D-HOPE in grafts from ECD resulted in donors in the D-HOPE group being older (72 vs. 68 years, *p* = 0.003) and with higher BMI (27 vs. 25, *p* = 0.001). There was also a trend towards a higher degree of macrovesicular steatosis in the D-HOPE group (3% vs. 2%, *p* = 0.07). As all DCD LTs were performed with sequential normothermic regional perfusion followed by D-HOPE, donor types were significantly different. Total preservation time was comparatively longer (474 vs. 404 min, *p* < 0.001) when D-HOPE was utilized, as the transplant operations were scheduled to ensure a minimum perfusion time of 90 min.

**Figure 1:**
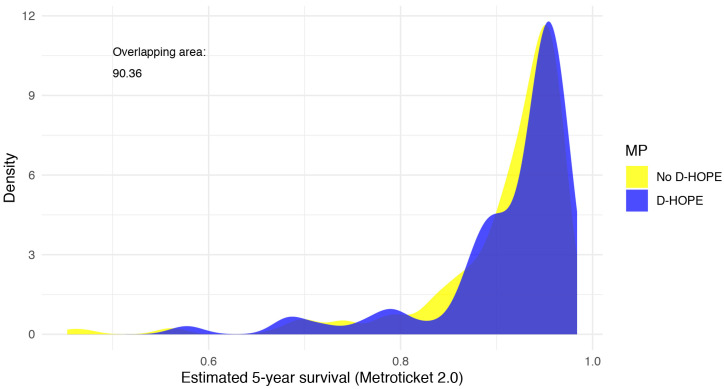
Kernel density plot showing comparable 5-year survival probability by preservation modality, as estimated using Metroticket 2.0 model.

HCC characteristics and pre-LT downstaging were similar between study groups, as well as 5-year RFS estimated by the Metroticket 2.0 model (Figure 1).

**Figure 2:**
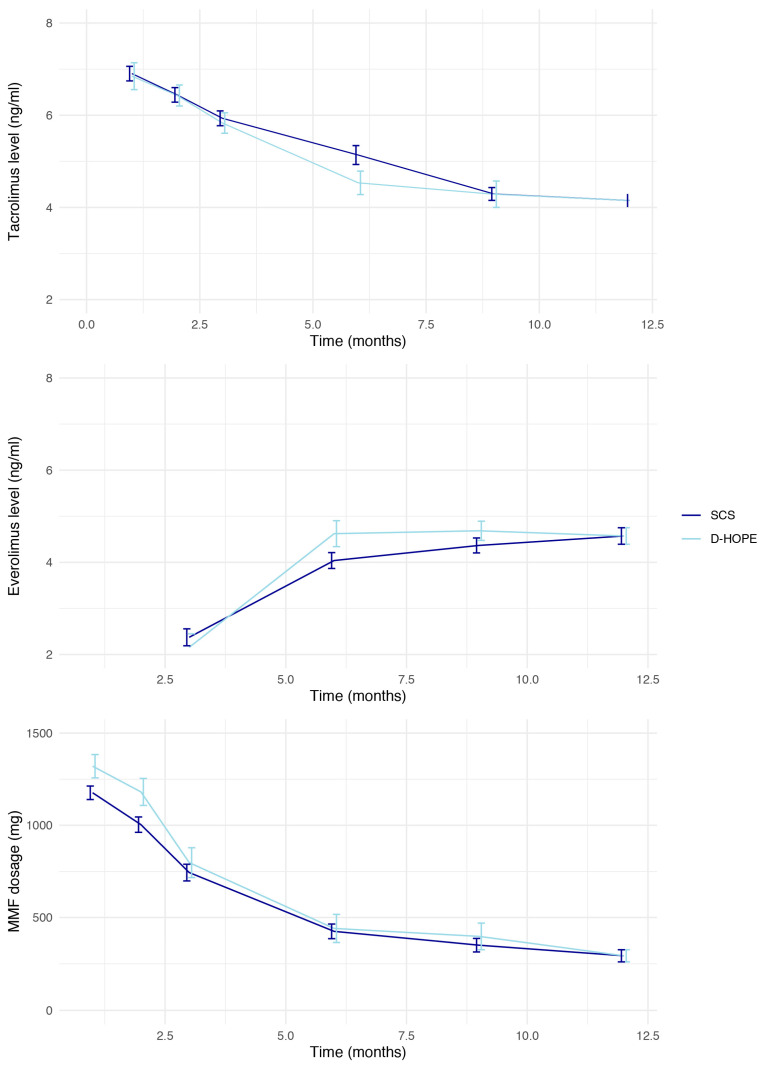
Immunosuppression levels and dosage according to preservation modality in the 12 months after transplant. Lines represent mean values, whereas vertical error bars represent standard error.

Immunosuppression management was comparable between study groups, except that patients in the D-HOPE group were more frequently administered basiliximab as an induction therapy (53% vs. 20%, *p* < 0.001). Everolimus was introduced in 71% of patients in each group. No differences were observed in terms of exposure to tacrolimus or everolimus, or mycophenolate mofetil dosage (Figure 2).

### 3.1. HCC Recurrence and Postoperative Outcomes

The HCC recurrence rate was comparable between study groups, with 10% and 8.9% of patients in the D-HOPE and SCS group experiencing recurrence (*p* = 0.95) (Table 2). Peak AST and ALT levels were lower in the D-HOPE group compared to the SCS group (AST: 903 vs. 1140, *p* = 0.022; ALT: 496 vs. 792, *p* = 0.002, respectively). Other considered outcomes were comparable between groups shown, including early (10% vs. 10%, *p* = 1) and late (4.5% vs. 3%, *p* = 0.145) rejection rates.

Significant predictors of HCC recurrence were identified through univariable and multivariable Cox regression analysis (Table 3). Evidence of microvascular invasion at explant pathology and HCC grading G3–G4 were the only variables independently affecting RFS, at both univariable and multivariable Cox regression. The use of D-HOPE did not influence HCC recurrence.

To obtain more robust estimates of the variables affecting recurrence, a further analysis using Bayesian model averaging was performed, confirming the absence of a significant association between D-HOPE and HCC recurrence (percentage of inclusion = 13%, hazard ratio = 1, credibility interval = 0.53–3.57). Microvascular invasion and G3–G4 grading were confirmed as the variables with the highest percentage of inclusion among predictive models and thus more significantly associated with HCC recurrence (percentage of inclusion = 100%, hazard ratio = 5, credibility interval = 2.09–12.4 and percentage of inclusion = 97%, hazard ration = 3, credibility interval = 1.32–8.00, respectively) (Table 4).

### 3.2. Survival Analysis

**Figure 3:**
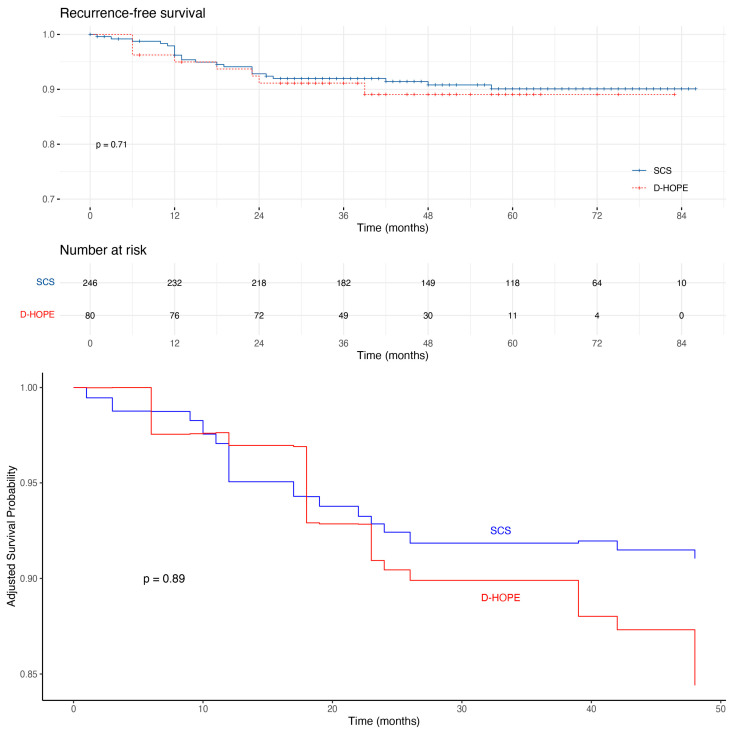
Raw and IPTW-adjusted Kaplan–Meier curves for recurrence free-survival according to preservation modality.

Median follow-up was 59 (36–72) and 40 (32–52) months in SCS and D-HOPE groups, respectively. Survival analysis showed comparable RFS rates between study groups (log-rank *p* = 0.71). Median (95% confidence interval) 1-year RFS was 96% (94–99%) and 95% (90–100%) in the SCS and D-HOPE groups, respectively (Figure 3). To account for potential confounders, IPTW-adjusted RFS analysis was performed, including donor age, donor BMI, macrovesicular steatosis, cold ischemia time, recipient age, HCC grading, and microvascular invasion as covariates. These variables were selected due to their association with HCC recurrence or unbalance between study groups. Even after adjustment, RFS did not significantly differ between study groups (*p* = 0.89) (Figure 3, second panel).

## 4. Discussion

Recent advancements in HCC staging have improved access to LT for patients suffering from HCC [7]. HCC recurrence rate after LT varies between 6% and 24% [14,67], and it is largely determined by tumor burden and biology.

A large body of preclinical studies has shown an association between IRI and tumor recurrence after LT (Table 5). The mechanisms lays in the inflammatory response triggered by liver IRI, which creates a favorable local microenvironment enhancing tumor cells’ invasiveness [11,12,13,14,15,16,17,18,19,20,21,22,23,24,25,26,27,28,29,30,68]. Sinusoidal dysfunction sustains tissue hypoxia and triggers the activation of the hypoxia-inducible factor 1α pathway and its downstream genes, leading to neo-angiogenesis and apoptosis inhibition [15,16,24]. The overexpression of proliferation regulators, such as Rho-family proteins, and adhesion molecules, such as E-selectin, sustains tumor cell growth and their migration into the extravascular space [11,12,25,26,28]. C-X-C motif chemokine ligand 10 (CXCL10), a chemoattractant, promotes macrophage activation and the recruitment of endothelial progenitor cells (EPC) into the liver [13,14,22,30]. Both CXCL10 and EPC circulating levels were increased in patients with HCC recurrence and were associated with neoangiogenesis and invasiveness [14]. Moreover, CXCL10 and matrix metalloproteinases, activated in response to IRI, enhance regulatory T cells migration and consequent immune response suppression, which ultimately supports tumor growth [13,27,29].

Several clinical studies [14,55,67,69,70,71,72,73,74,75,76] have investigated the association between graft quality and HCC recurrence (Table 6). Small-for-size liver grafts are exposed to a transient hemodynamic stress that aggravates sinusoidal damage and consequently increases IRI, possibly contributing to the higher HCC recurrence in living donor LT recipients [14,76]. Orci et al. [73] reported an increased risk of recurrence in patients receiving a graft from elderly (aged > 60 years), diabetic, obese (BMI > 35), or severely steatotic donors. Prolonged donor or recipient warm ischemia time have been identified as independent risk factors for HCC recurrence in multiple series [70,72,73], but the evidence concerning the association between the utilization of livers from DCD donors and HCC recurrence is conflicting, with some studies suggesting a higher risk [67] and others reporting similar outcomes [74].

The utilization of ECD organs has highlighted the limitations of SCS and has reignited interest in dynamic organ preservation techniques due to their ability to attenuate IRI [77]. Specifically, HOPE enables controlled tissue reoxygenation and prevents mitochondrial respiratory chain dysfunction, reducing IRI [54]. In contrast, normothermic machine perfusion (NMP) relies on reproducing a physiological environment that maintains liver metabolism and prevents ATP depletion to protect the organ [54]. Given the association between IRI and HCC recurrence, the use of machine perfusion could potentially improve outcomes of LT for HCC. However, pre-clinical studies are limited and have failed to provide solid evidence to support this hypothesis [20].

A recent clinical study by Mueller et al. [55] compared 70 DCD grafts treated with end-ischemic HOPE with 70 DBD grafts preserved with SCS, showing lower HCC recurrence rate in the HOPE group (5.7% vs. 25.7%, *p* = 0.002), despite the utilization of high-risk DCD grafts. Concerning NMP, the ischemia-free liver transplantation (IFLT) protocol developed by the Guangzhou group in China has shown promising preliminary results [69]. A propensity score-matched analysis showed better recurrence-free survival at 1 and 3 years for recipients with HCC after IFLT as compared to conventional LT (92% vs. 73%, *p* = 0.006 and 87% vs. 46.3%, *p* = 0.048, respectively) [69].

In our single-center study, we analyzed a cohort of 326 liver transplants in patients with HCC to investigate whether D-HOPE may improve recurrence-free survival (RFS). The lower number of patients receiving a D-HOPE-treated graft was determined by its use in selected cases based on donor and recipient characteristics and expected preservation time. Our patient population was homogeneous in terms of recipient and HCC characteristics, operational variables, and postoperative management. In particular, the management of immunosuppression, which could act as an important confounder [78,79], was similar, with a therapeutic switch to everolimus performed in most cases. The only difference in immunosuppression management was represented by a more deliberate use of induction therapy with basiliximab in the D-HOPE group. However, conflicting results have been reported regarding a potential influence of induction by basiliximab on HCC recurrence after liver transplantation [80,81], and these have not been confirmed in the setting of a randomized trial. D-HOPE-treated grafts were procured from older donors with higher BMI, reflecting the preferential allocation of grafts from ECD to D-HOPE group. Furthermore, 14 (17%) grafts in the D-HOPE group were procured from DCD donors. Despite these differences, D-HOPE-treated grafts showed lower AST and ALT peaks, in keeping with lower IRI after D-HOPE use [54]. All other analyzed outcomes were comparable, suggesting that D-HOPE treatment compensated for the features of marginality of the donors in this group.

Despite a thorough analysis, we were unable to demonstrate a significant impact of D-HOPE on HCC recurrence. In our series, HCC recurrence was closely associated with HCC features, such as microvascular invasion and tumor grading, which is consistent with the evidence from the literature [82,83]. In contrast with the findings from the Zurich group [55], even after adjusting for potential confounders, we did not observe any significant differences in HCC recurrence rate between recipients of a D-HOPE-treated graft and those who received a graft preserved by SCS. Contrary to the aforementioned study, where the DBD cohort had a high HCC recurrence rate (25.7%), in our cohort, we observed a consistently lower HCC recurrence rate (9% vs. 10%) across study groups. Interestingly, no recipient of a DCD graft procured using sequential NRP + D-HOPE [48,64] developed recurrence. However, the small sample size of this group and the lack of a comparator (SCS alone is not allowed for DCD donors in Italy) did not allow for a focused analysis.

Some limitations of our study, possibly explaining the apparent lack of effect of D-HOPE on HCC recurrence, must be acknowledged. First, given the relatively low incidence of HCC recurrence after LT and the predominant effect of HCC features in determining recurrence, a larger sample size could be required to demonstrate the effect of any protective intervention. Second, our cohort predominantly included grafts from DBD donors, which are less exposed to severe IRI than those from DCD donors. Thus, the effect of a technique that can potentially reduce HCC recurrence rate by mitigating IRI could be more evident in a higher-risk setting such as DCD LT. Third, in our cohort D-HOPE was preferentially, although not invariably, used in grafts from ECD donors. Given the retrospective nature of our study, it is possible that the selection bias, despite adjusting for potential confounders, could have masked the protective effects of D-HOPE towards HCC recurrence.

In conclusion, our experience did not show a significant impact of end-ischemic D-HOPE on HCC recurrence after LT. Limited sample size, retrospective design, and the preferential use of D-HOPE in ECD grafts represent the main limitations of the study. Nevertheless, our results corroborate the fundamental role of D-HOPE in expanding the donor pool and improving access to LT for patients suffering from HCC, as outcomes were similar in the two groups despite the marginal characteristics of the grafts treated with D-HOPE. The impact of D-HOPE on HCC recurrence in LT should be investigated in the setting of a randomized controlled trial with a larger sample size.

## Figures and Tables

**Table 1:** Baseline features in the whole cohort and according to preservation modality (SCS versus D-HOPE).

		Overall (n = 326)	SCS (n = 246)	D-HOPE (n = 80)	*p* Value
**Recipient and donor features**					
Recipient age		59.0 [55.3, 63.4]	58.9 [55.1, 62.8]	60.1 [55.7, 63.6]	0.147
Recipient gender	F	52 (16)	41 (17)	11 (14)	0.658
	M	274 (84)	205 (83)	69 (86)	
Indication	Alcoholic cirrhosis	55 (17)	41 (17)	14 (18)	0.668
	Autoimmune hepatitis	2 (1)	2 (1)	0 (0)	
	Cholestatic liver disease	4 (1)	2 (1)	2 (2)	
	NASH	12 (4)	8 (3)	4 (5)	
	Viral hepatitis	222 (68)	171 (70)	51 (64)	
	Other	31 (10)	22 (9)	9 (11)	
Waiting time (days)		35.0 [16.5, 90.5]	35.0 [17.0, 86.0]	32.0 [14.5, 94.5]	0.713
Recipient BMI		25.8 [23.5, 28.1]	25.7 [23.4, 28.3]	26.0 [23.9, 27.8]	0.548
MELD		10.0 [8.0, 14.0]	10.0 [8.0, 14.0]	10.0 [8.0, 14.0]	0.872
Prev. abdominal surgery		127 (39)	90 (37)	37 (46)	0.159
Donor age		68.7 [57.7, 77.4]	67.9 [55.7, 76.5]	71.8 [60.7, 82.4]	0.003
Donor gender	F	134 (41)	105 (43)	29 (36)	0.376
	M	192 (59)	141 (57)	51 (64)	
Donor type	DBD	312 (96)	246 (100)	66 (82)	<0.001
	DCD cat. II	1 (0)	0 (0)	1 (1)	
	DCD cat. III	13 (4)	0 (0)	13 (16)	
Donor BMI		25.7 [23.5, 28.4]	25.4 [23.0, 27.7]	27.2 [24.5, 29.4]	0.001
Macrosteatosis %		2.0 [0.0, 10.0]	2.0 [0.0, 10.0]	3.0 [0.0, 10.0]	0.070
Macrosteatosis ≥ 15%		57 (18)	39 (16)	18 (22)	0.266
D-MELD		674 [537, 916]	659 [527, 897]	715 [558, 968]	0.078
BAR		5.0 [3.0, 5.0]	5.0 [3.0, 5.0]	5.0 [3.0, 5.0]	0.113
DRI		1.8 [1.4, 2.3]	1.7 [1.4, 2.3]	2.1 [1.4, 2.4]	0.137
Total preservation time (min)		417 [364, 471]	403 [354, 452]	474 [411, 519]	<0.001
Rec. warm ischemia time (min)		23 [20, 27]	23 [21, 27]	22 [20, 27]	0.300
D-HOPE time (min)		0 [0, 0]	0 [0, 0]	144 [117, 180]	<0.001
**HCC characteristics**					
N. nodes at LT	0	3 (1)	1 (0)	2 (2)	0.322
	1	133 (41)	99 (40)	34 (42)	
	2–3	92 (28)	73 (30)	19 (24)	
	3–4	67 (21)	48 (20)	19 (24)	
	≥5	31 (10)	25 (10)	6 (8)	
Max diam. at LT (mm)		20.0 [14.0, 30.0]	20.0 [15.0, 30.0]	20.0 [14.0, 30.0]	0.901
Tot. diam. at LT (mm)		32.0 [21.0, 50.0]	34.0 [21.0, 52.0]	30.0 [18.8, 48.0]	0.298
AFP at LT (ng/mL)		4.5 [2.9, 11.7]	4.5 [2.9, 12.2]	4.3 [3.0, 8.8]	0.776
Estimated 5-year survival *		0.9 [0.9, 1.0]	0.9 [0.9, 1.0]	0.9 [0.9, 1.0]	0.232
Estimated 5-year survival *	<80%	28 (9)	20 (9)	8 (11)	
	80–85%	12 (4)	11 (5)	1 (1)	
	85–90%	46 (15)	31 (14)	15 (20)	
	90–95%	109 (37)	89 (40)	20 (27)	
	>95%	103 (35)	72 (32)	31 (41)	0.118
Downstaging (Y/N)		264 (81)	201 (82)	63 (79)	0.673
Downstaging (n. procedures)		1.0 [1.0, 2.0]	1.0 [1.0, 2.0]	1.0 [1.0, 2.0]	0.605
Locoregional		247 (76)	191 (78)	56 (70)	0.217
SBRT		33 (10)	19 (8)	14 (18)	0.021
Liver resection		16 (5)	13 (5)	3 (4)	0.799
AFP maximum level (ng/mL)		7.0 [3.5, 26.4]	7.0 [3.2, 28.2]	6.7 [3.9, 22.9]	0.894
N. nodes at pathology	0	3 (1)	2 (1)	1 (1)	
	1	111 (34)	88 (36)	23 (29)	
	2–3	78 (24)	57 (23)	21 (27)	
	3–4	74 (23)	51 (21)	23 (29)	
	≥5	56 (17)	45 (19)	11 (14)	0.452
Max diam. pathology (mm)		25.0 [18.0, 35.0]	25.0 [18.0, 35.5]	26.0 [17.5, 34.0]	0.827
Tot. diam. pathology (mm)		42.0 [28.0, 63.0]	40.0 [29.2, 61.8]	45.0 [25.5, 73.0]	0.627
Grading	G1–G2	174 (72)	136 (72)	38 (69)	0.764
	G3–G4	69 (28)	52 (28)	17 (31)	
Microvascular invasion (%)		55 (17)	44 (18)	11 (14)	0.502
**Immunosuppression and rejection**					
Induction (basiliximab)		93 (29)	50 (20)	43 (54)	<0.001
TAC start day	0	49 (38)	34 (40)	15 (33)	
	1	46 (35)	32 (38)	14 (30)	
	2	19 (15)	9 (11)	10 (22)	
	3	7 (5)	5 (6)	2 (4)	
	4	6 (5)	3 (4)	3 (7)	
	5	2 (2)	1 (1)	1 (2)	
	7	1 (1)	0 (0)	1 (2)	0.405
12-month TAC AUC (mg)		55.1 [41.6, 69.5]	55.6 [42.9, 69.6]	49.3 [40.0, 68.8]	0.307
MMF mean dose		395 [166, 875]	416 [166, 875]	375 [250, 916]	0.324
Switch to EVE (Y/N)		218 (71)	165 (71)	53 (71)	1.000
12-month EVE AUC (mg)		40.4 [23.9, 54.6]	40.1 [22.2, 54.9]	43.2 [32.1, 52.2]	0.640
Early rejection (y/n)		32 (10)	24 (10)	8 (10)	1.000
Steroid pulses		29 (9)	23 (9)	6 (8)	0.780
Thymoglobulin		2 (1)	2 (1)	0 (0)	1.000
Late rejection		18 (6)	10 (4)	8 (11)	0.079

* According to Metroticket 2.0 model. Data are presented as number (percentage) or median (interquartile range), as appropriate. Abbreviations: AFP, alpha fetoprotein; BAR, balance of risk score; BMI, body mass index; D-HOPE, dual-hypothermic oxygenated machine perfusion; DBD, donation after brain death; DCD, donation after cardiac death; D-MELD, donor age * MELD; DRI, donor risk index score; EVE, everolimus; HCC, hepatocellular carcinoma; LT, liver transplantation; MELD, model for end-stage liver disease; MMF, mycophenolate mofetil; RAI, rejection activity index; SBRT, stereotactic body radiotherapy; TAC, tacrolimus.

**Table 2:** Postoperative outcomes.

		Overall (n = 326)	SCS (n = 246)	D-HOPE (n = 80)	*p* Value
HCC recurrence		30 (9)	22 (9)	8 (10)	0.951
AST peak		1089.0 [671.0, 1782.0]	1140.0 [719.0, 1831.0]	903.0 [561.8, 1570.0]	0.022
ALT peak		699.0 [420.0, 1135.0]	742.0 [454.0, 1163.0]	496.5 [273.8, 984.2]	0.002
EAD		87 (27)	66 (27)	21 (26)	1.000
AKI stage	no	120 (37)	93 (38)	27 (34)	0.081
	1	126 (39)	90 (37)	36 (45)	
	2	56 (17)	48 (20)	8 (10)	
	3	24 (7)	15 (6)	9 (11)	
Complications (Clavien–Dindo)	0	32 (10)	18 (7)	14 (18)	0.143
	1	75 (23)	61 (25)	14 (18)	
	2	167 (51)	127 (52)	40 (50)	
	3a	6 (2)	5 (2)	1 (1)	
	3b	23 (7)	17 (7)	6 (8)	
	4a	15 (5)	12 (5)	3 (4)	
	4b	4 (1)	2 (1)	2 (2)	
	5	4 (1)	4 (2)	0 (0)	
Clavien–Dindo ≥ 3 complications		52 (16)	40 (16)	12 (15)	0.927
CCI at discharge		20.9 [8.7, 29.6]	20.9 [8.7, 29.6]	20.9 [8.7, 29.6]	0.243
ICU stay (days)		3.0 [2.0, 4.0]	3.0 [2.0, 4.0]	3.0 [2.0, 5.0]	0.720
Hospital stay (days)		10.0 [8.0, 15.8]	10.0 [8.0, 14.8]	11.0 [8.0, 18.0]	0.967
Biliary complications (overall)		63 (19)	49 (20)	14 (18)	0.754
Anastomotic complications		58 (18)	44 (18)	14 (18)	1.000
Ischemic cholangiopathy		7 (2)	7 (3)	0 (0)	0.280

Data are presented as number (percentage) or median (interquartile range), as appropriate. Abbreviations: AKI, acute kidney injury; ALT, alanine aminotransferase; AST, aspartate aminotransferase; CCI, comprehensive complication index; D-HOPE, dual-hypothermic oxygenated machine perfusion; EAD, early allograft dysfunction; HCC, hepatocellular carcinoma.

**Table 3:** Univariable and multivariable Cox regression analysis of variables associated with recurrence-free survival.

	Univariable Analysis		Multivariable Analysis	
	HR (95% CI for HR)	*p* Value	HR (95% CI for HR)	*p* Value
N. nodes at LT	1.1 (0.94–1.4)	0.17		
Max diam. at LT	1 (0.98–1)	0.85		
AFP at TL	1 (1–1)	0.21		
Downstaging	0.72 (0.31–1.7)	0.45		
Grading G3–G4	5 (2.1–12)	<0.001	3.2 (1.3–7.9)	0.10
Microvascular invasion	5.2 (2.6–11)	<0.001	5 (2–11.9)	<0.001
Donor age (years)	1 (0.98–1)	0.66		
Donor BMI	0.98 (0.91–1.1)	0.7		
DCD donor	3.9 × 10^−8^ (0-Inf)	1		
D-HOPE	1.2 (0.52–2.6)	0.71	1.34 (0.5–3.4)	0.54
PRBC transfusion (units)	0.98 (0.92–1)	0.57		
Lactate end of LT (mmol/L)	1.1 (0.88–1.5)	0.32		
Severe PRS	0.5 (0.12–2.1)	0.35		
AST peak (IU/L)	1 (1–1)	0.32		
ALT peak (IU/L)	1 (1–1)	0.9		
L-GrAFT (risk %)	1 (0.99–1)	0.2		
CCI at discharge	0.99 (0.97–1)	0.66		
Tacrolimus AUC (mg)	1 (0.97–1)	0.74		
Everolimus AUC (mg)	0.99 (0.97–1)	0.41		

Abbreviations: AFP, alpha fetoprotein; ALT, alanine aminotransferase; AST, aspartate aminotransferase; CCI, comprehensive complication index; DCD, donation after cardiac death; L-GrAFT, liver graft assessment following transplantation score; LT, liver transplantation; PRBC, packed red blood cells; PRS, post-reperfusion syndrome.

**Table 4:** Bayesian model averaging for HCC recurrence.

	p Inclusion	HR	CI 95%	pd
Microvascular invasion	100.0	5.08	2.09; 12.362	1.00
Grading G3–G4	97.2	3.25	1.323; 7.997	0.99
DCD donor	18.4	0.00	0; Inf	0.50
Macrovesicular steatosis (%)	17.5	0.97	0.917; 1.031	0.83
L-GrAFT score	16.4	1.01	0.989; 1.035	0.84
D-HOPE	13.3	1.37	0.528; 3.568	0.74
Donor age (years)	12.9	1.01	0.982; 1.034	0.72
Donor gender (male)	10.6	0.82	0.345; 1.938	0.68
Cold ischemia time (min)	9.3	1.00	0.996; 1.006	0.62
Recipient BMI	9.1	1.01	0.891; 1.157	0.59
Packed red blood cells transfusion (units)	8.5	1.01	0.955; 1.067	0.63
Donor BMI	8.2	1.00	0.919; 1.097	0.54

Abbreviations: AFP, alpha fetoprotein; ALT, alanine aminotransferase; AST, aspartate aminotransferase; CCI, comprehensive complication index; DCD, donation after cardiac death; L-GrAFT, liver graft assessment following transplantation score; LT, liver transplantation; PRBC, packed red blood cells; PRS, post-reperfusion syndrome.

**Table 5:** Preclinical studies investigating the association between liver IRI and tumor progression.

Author, Year	Animal	Model	Ischemia	Tumor	Findings
Doi et al., 2002 [11]	Rat	Partial IRI	30 vs. 60 min	Colorectal liver metastases	▪ IRI enhanced tumor growth ▪ ↑ E-selectin in ischemic livers
Doi et al., 2002 [68]	Rat	Partial IRI	60 min	Colorectal liver metastases	▪ IRI enhanced tumor growth ▪ neutrophil elastase inhibition reduced the number of hepatic metastases
Yoshida et al., 2003 [12]	Rat	Partial IRI vs. intermittent clamping	60 min	Colorectal liver metastases	▪ IRI enhanced tumor growth in both ischemic and non-ischemic lobes ▪ Intermittent clamping reduced the number of hepatic metastases and E-selectin expression
van der Bilt et al., 2005 [23]	Mouse	Partial IRI	45 min	Colorectal liver metastases	▪ Tumor growth was markedly stimulated in ischemic lobes vs. non-ischemic lobes ▪ Intermittent clamping completely prevented IRI-stimulated tumor growth ▪ Ischemic preconditioning, α-tocopherol and ascorbic acid failed to protect against IRI-stimulated tumor growth
van der Bilt et al., 2007 [24]	Mouse	Partial IRI	45 min	Colorectal liver metastases	▪ IRI-stimulated tumor growth occurs preferentially in areas of tissue hypoxia, and elevated HIF-1α expression ▪ Reducing microcirculatory impairment with atrasentan and L-arginine as well as inhibiting HIF-1α with 17-DMAG resulted in reduced tumor growth
Ogawa et al., 2007 [25]	Rat	LT	-	HCC	▪ Tacrolimus activates Rho/ROCK signal pathway to enhance HCC cell migration ▪ ROCK inhibition suppresses HCC recurrence
Man et al., 2007 [26]	Rat	Partial IRI +/− major hepatectomy	60 min	HCC	▪ IRI and major hepatectomy enhanced tumor growth (↑ PCNA and VEGF) ▪ IRI and major hepatectomy promoted invasiveness by overexpressing Rho family genes (Rac1, ROCK, Cdc42) in tumor tissues
Nicoud et al., 2007 [27]	Mouse	Partial IRI	30 min	Colorectal liver metastases	▪ ↑ MMP9 in ischemic livers ▪ Doxycycline inhibits IRI-induced MMP9 and reduced tumor growth ▪ Inhibition of MMP9 reduced hepatic metastases
Man et al., 2008 [28]	Rat	Standard vs. small-for-size graft LT	-	HCC	▪ Small-for-size grafts had higher tumor growth, with increased angiogenesis (↑ VEGF) and invasiveness (ROCK)
Ushitora et al., 2009 [29]	Rat	LT	-	HCC	▪ Immunomodulation with FTY720 (Fingolimod) reduced tumor growth (↓ MAPK)
Man et al., 2010 [30]	Rat	Standard vs. small-for-size graft LT	-	HCC	▪ inflammatory chemokine CXCL10 was over-expressed in small-for-size liver grafts and their tumors ▪ CXCL10 promoted macrophage infiltration in the early phase after LT and tumor-associated macrophage activation in liver tumors developed in the late phase after LT
Li et al., 2012 [13]	Rat	Partial IRI +/− major hepatectomy	30 min	HCC	▪ Immunomodulation with FTY720 (Fingolimod) reduced tumor growth and lung metastases ▪ FTY720 reduced circulating and bone marrow EPCs and gene expressions of CXCL10, VEGF, CXCR3, CXCR4
Ling et al., 2014 [14]	Rat Mouse	Standard vs. small-for-size graft LT Partial IRI + major hepatectomy	45 min	HCC	▪ Small-for-size liver graft were associated with higher circulating EPCs and intragraft and plasma CXCL10/CXCR3 levels in tumor free LT model ▪ Both EPCs and CXCL10 injections promoted tumor growth, angiogenesis and lung metastasis
Oldani et al., 2014 [15]	Rat	DCD LT	10 or 30 min	HCC	▪ Donor ischemia increased tumor growth ▪ 2 h of in vivo NMP reduced tumor growth and modulated inflammatory genes expression (↓ Hmox1, HIF-1α, serpine1, ↑ IL10)
Hamaguchi et al., 2016 [16]	Rat	Major hepatectomy	5 vs. 10 vs. 15 min	HCC	▪ 15 min of intermittent clamping promoted tumor growth by inducing HIF-1α and IL-6–JAK–STAT3 signaling pathways
Orci et al., 2016 [17]	Mouse	Partial IRI	30 min	HCC	▪ Steatosis + IRI increased tumor growth ▪ Ischemic preconditioning reduced tumor load in steatotic livers exposed to IRI
Wang et al., 2017 [18]	Rat Mouse	DCD LT Partial IRI	30 min 15 vs. 30 vs. 60 min	HCC	▪ PARP-1 inhibition reduced HCC recurrence in both LT and partial IRI models ▪ PARP-1-induced susceptibility to recurrence was mediated by CXCL1/CXCR2 signaling and consequent neutrophil and angiogenesis activation
Orci et al., 2018 [19]	Mouse	Partial IRI	60 min	HCC	▪ Ischemic preconditioning, gut decontamination with antibiotics and pharmacological TLR4 inhibition reduced tumor growth
Oldani et al., 2019 [20]	Rat	DCD LT	60 min	HCC	▪ Donor ischemia increased tumor growth ▪ Both HOPE and NMP failed to bring a measurable benefit in terms of cancer implantation/growth reduction
Yang et al., 2019 [21]	Mouse	Partial IRI	60 min	HCC	▪ Steatosis + IRI increased tumor growth ▪ ALOX12–12-HETE pathway inhibition reduced HCC recurrence
Li et al., 2020 [22]	Rat	Partial IRI +/− major hepatectomy	30 min	HCC	▪ Immunomodulation with FTY720 (Fingolimod) reduced tumor growth, lung metastases and circulating Tregs ▪ FTY720 combined with rapamycin further suppressed tumor growth and invasiveness

Abbreviations: ↑, increase; CXCL10, C-X-C motif chemokine ligand 10; DCD, donation after cardiac death; EPC, endothelial progenitor cells; HCC, hepatocellular carcinoma; HIF-1α, hypoxia-inducible factor 1α; Hmox1, heme oxygenase (decycling) 1; HOPE, hypothermic oxygenated perfusion; IRI, ischemia/reperfusion injury; LT, liver transplantation; MAPK, mitogen-activated protein kinase; MMP9, matrix metalloproteinase 9; NMP, normothermic machine perfusion; PARP-1, poly (ADP-ribose) polymerase; PCNA, proliferating cell nuclear antigen; ROCK, Rho/Rho-associated kinase; VEGF, vascular endothelial growth factor.

**Table 6:** Clinical studies investigating the association between liver IRI and HCC recurrence after LT.

Author, Year	Study	n	Donor	Intervention	Recurrence	Findings
Ling et al., 2014 [14]	Retrospective, single center	115	DBD	Standard (n = 37) vs. small-for-size graft (n = 78) LT	8% vs. 24.4%	Patients with small-for-size liver graft had higher HCC recurrence, accompanied with increased circulating EPCs and CXCL10 levels
Kornberg et al., 2015 [70]	Retrospective, single center	103	DBD	-	23.3%	WIT > 50 min identified as an independent predictor of HCC recurrence
Kornberg et al., 2015 [71]	Retrospective, single center	106	DBD	Post-operative PGE1 therapy	23.6%	- PGE1 therapy identified as an independent prognostic factor for early HCC recurrence (within 12 months) - PGE1 therapy identified as an independent prognostic factor for recurrence-free survival in Milan-out patients
Nagai et al., 2015 [72]	Retrospective, multicenter	391	DBD	-	15.3%	CIT > 10 h and WIT > 50 min identified as independent predictors of HCC recurrence
Orci et al., 2015 [73]	Retrospective, UNOS registry	9724	DBD DCD	-	-	Donor age > 60 y, BMI > 35, diabetes, steatosis > 60% and WIT > 19 min associated with an increased HCC recurrence risk
Khorsandi et al., 2016 [74]	Retrospective, single center	347	DBD DCD	-	12.1%	DCDs had same HCC recurrence rates than DBDs
Grat et al., 2018 [75]	Retrospective, single center	195	DBD	-	13.8%	Post-reperfusion AST < 1896 U/L and LDH < 4670 U/L increased HCC recurrence-free survival after LT in Milan-in patients
Silverstein et al., 2020 [67]	Retrospective, UNOS registry	7563	DBD DCD	-	6.4% vs. 7.6%	DCD donor was an independent predictor of post-LT mortality. After stratifying for risk of HCC recurrence, only subgroups at higher risk for HCC recurrence had lower survival rates.
Mueller et al., 2020 [55]	Retrospective, multicenter	280	DBD DCD	HOPE- treated DCDs vs. SCS- DCDs/DBDs	5.7% (DCD-HOPE, center A); 25.7% (DBD, center A); 14.3% (DCD, center B); 17.1% (DBD, center B)	DCD grafts exposed to 2 h of end-ischemic HOPE had lower HCC recurrence compared to cold-stored DBD grafts from the same center
Liu et al., 2021 [76]	Retrospective, single center	329	DBD	Standard (n = 149) vs. small-for-size graft (n = 180) LT	10% vs. 19.4%	Patients with small-for-size liver graft had higher HCC recurrence, accompanied with increased circulating MDSCs and CXCL10 levels
Tang et al., 2021 [69]	Retrospective, single center	226	DBD	Ischemia-free LT (n = 30) vs. SCS-LT (n = 196)	-	Ischemia-free LT was associated with higher HCC recurrence-free survival rates than conventional LT

Abbreviations: CIT, cold ischemia time; CXCL10, C-X-C motif chemokine ligand 10; DBD, donation after brain death; DCD, donation after cardiac death; EPC, endothelial progenitor cells; HCC, hepatocellular carcinoma; HOPE, hypothermic oxygenated perfusion; MDSC, myeloid-derived sup- pressor cells; PGE1, prostaglandin E1; SCS, static cold storage; WIT, warm ischemia time.

## Data Availability

Research data will not be made publicly available due to privacy reasons.
